# Addressing the Needs of Persons Living With Dementia Across Care Settings (A Collaborative Symposium between the Assisted Living and Research in Quality Care Interest Groups)

**DOI:** 10.1093/geroni/igaa057.2314

**Published:** 2020-12-16

**Authors:** Howard Degenholtz, Candace Kemp, Doug Pace

## Abstract

Understanding the needs of persons living with dementia is critical to promoting quality of life and care. Co-sponsored by the Assisted Living (AL) and Research in Quality Care Interest Groups, this symposium includes four papers that present new ways to use claims data, qualitative data, benchmarking data, and intervention data to promote well-being for persons living with dementia. First, Degenholtz and Van Cleve use Pennsylvania Medicaid data from 2014-2016 to examine the provision of personal care to persons with and without dementia. They find that those living with dementia received more personal care per day across levels of physical disability and discuss implications for home and community based services policy. Next, Kemp et al. investigate meaningful engagement among assisted living residents with dementia using qualitative data collected over a one-year period in four diverse care communities. Findings show a range of engagement experiences and point to the influence of key resident, care partner, and care community influences. Third, Morgan et al. use data from a statewide probability sample of nursing home staff (n=438) to identify barriers and facilitators to person-centered care. Findings show key barriers to delivering person-centered care, including a lack of staff empowerment practices and low use of consistent assignment. Last, Zimmerman et al. present an evidence-based program, “Mouth Care Without a Battle” developed in nursing homes. Using data from over 2,000 assisted living stakeholders, they situate their findings within implementation science and the NIH Stage Model and make their findings transferable regardless of focus or setting.

